# Debate: Lipid-lowering Therapies and Diabetes Development

**DOI:** 10.1007/s11883-024-01270-y

**Published:** 2025-01-08

**Authors:** Julia Brandts, Dirk Müller-Wieland

**Affiliations:** 1 Department of Internal Medicine I, University Hospital Aachen, Pauwelsstraße, 30 52074 Aachen, Germany; 2 Imperial Centre for Cardiovascular Disease Prevention, School of Public Health, Imperial College London, London, UK

**Keywords:** Statins, Diabetes mellitus, Statins, Lipid-lowering therapies, Cardiovascular risk, LDL-cholesterol

## Abstract

**Purpose of Review:**

This review explores the relationship between lipid-lowering therapies, particularly statins, and the risk of new-onset diabetes (NOD). It examines the underlying mechanisms and evaluates whether other lipid-lowering agents present similar risks.

**Recent Findings:**

Recent meta-analyses further underscore a dose-dependent increase in NOD risk with statin therapy, particularly with high-intensity statins. In contrast to other LDL-cholesterol lowering drugs and their impact on lipid metabolism in the liver, genetic and experimental studies indicate that statins may impair insulin secretion through various mechanisms, including alterations in small G protein function, calcium signaling, and cholesterol homeostasis in pancreatic beta cells. This might contribute to the increased risk of NOD.

**Summary:**

Statins effectively reduce cardiovascular events but increase the risk of NOD, potentially via intracellular pathways affecting liver and beta-cell function. Despite the cardiovascular benefits of statins, personalized treatment strategies and alternative lipid-lowering therapies may offer safer options for patients at risk of diabetes, potentially shaping future clinical guidelines and therapeutic approaches.

## Introduction

Statins are a cornerstone in the management of dyslipidemia and the prevention of atherosclerotic cardiovascular disease (ASCVD). Their role is especially critical for patients at increased risk of ASCVD events, including those with prediabetes and diabetes, due to their potent lipid-lowering effects and additional cardiovascular benefits. Numerous large-scale randomized controlled trials (RCTs) have demonstrated that statins effectively lower LDL-C levels by 20–50%, depending on the dose and specific statin used. This reduction in LDL-C is associated with a proportional reduction in the risk of major cardiovascular events with a 22% relative risk reduction per mmol/l LDL-C reduction[1].

Statins lower LDL-C levels and provide a range of pleiotropic effects, including anti-inflammatory and plaque-stabilizing properties, which are particularly beneficial in high-risk patients with atherosclerotic manifestations. These beneficial effects appear to be primarily related to effective LDL reduction and, therefore, are true for all LDL-C lowering therapies.

Despite this, however, an increased risk of new-onset diabetes (NOD) has been consistently reported for statins but not other lipid-lowering drugs, and therefore, we focus on this issue in the following short review.

## First Observations and Meta-Analysis

The first indication of an increased rate of NOD was observed in the JUPITER trial[2]. This was the initial randomized controlled trial (RCT) comparing rosuvastatin with a placebo, revealing a higher incidence of physician-reported diabetes in the rosuvastatin group (270 cases) compared to the placebo group (216 cases), with a statistically significant difference (P=0.01). It is important to note that these events were not adjudicated by the end-point committee.

In the trial, a small but statistically significant increase in physician-reported diabetes cases and median glycated hemoglobin (HbA1c) values was detected among those taking rosuvastatin. However, protocol-specified measurements in the JUPITER trial showed no significant differences in fasting blood glucose levels or glycosuria between the rosuvastatin and placebo groups during the follow-up period. Therefore, while the increase in diabetes reports among the rosuvastatin group might be due to chance, further investigation is necessary to confirm or refute a causal relationship. The non-adjudicated nature of physician-reported diabetes cases calls for a careful review of participants’ records for a better understanding of this potential effect.

Contrary to the JUPITER findings, earlier studies like the West of Scotland Coronary Prevention Study (WOSCOPS) provided insights into the effects of pravastatin on diabetes risk over 3.5 to 6.1 years of follow-up[3]. This study concluded that pravastatin therapy led to a 30% reduction in diabetes risk (P=0.042).

To resolve these mixed outcomes, a meta-analysis supported the JUPITER results [4]. This meta-analysis reviewed 13 statin trials involving 91,140 participants, of whom 4,278 developed diabetes (2,226 on statins and 2,052 on control treatment) over an average of four years. Statin therapy was associated with a 9% increased risk of incident diabetes (odds ratio [OR] 1.09; 95% CI 1.02–1.17), with minimal heterogeneity (I^2=11%) between trials. Meta-regression analysis revealed that the risk of developing diabetes was highest in trials involving older participants, while baseline body mass index (BMI) and changes in LDL-cholesterol levels did not significantly account for the variation in risk. It was estimated that treating 255 patients with statins for four years would result in one additional case of diabetes (95% CI 150–852).

Supporting these findings, observational data from the Women’s Health Initiative (WHI) focused on postmenopausal women further elucidated the association between statin use and NOD[5]. The WHI study, involving over 153,000 women, found that statin use at baseline was linked to a 71% increase in the risk of developing diabetes. This association persisted even after adjusting for various confounders and was consistent across different types of statins. These results underscored the potential class effect of statins in increasing diabetes risk and highlighted the importance of monitoring glucose levels in patients on long-term statin therapy.

## Meta-Analysis and Dose-/Intensity-Dependent Risk

In 2011, a further meta-analysis aimed to investigate the risk of NOD with intensive-dose statin therapy compared to moderate-dose treatment [6]. This meta-analysis included five trials with over 32,000 participants without diabetes at baseline and revealed that intensive-dose statin therapy was associated with a 12% increased risk of developing diabetes. The analysis indicated that intensive-dose therapy led to approximately two additional cases of diabetes per 1,000 patient-years compared to moderate-dose therapy. However, intensive-dose treatment also resulted in fewer cardiovascular events, illustrating the complex risk-benefit balance of statin therapy.

Additionally, a comprehensive network meta-analysis examined the dose-dependent nature of this risk across various types of statins[7]. This meta-analysis incorporated data from 17 randomized controlled trials covering over 113,000 patients and compared different statins and doses with placebo. The findings indicated that pravastatin was associated with the lowest risk of new-onset diabetes, while rosuvastatin and atorvastatin were linked to higher risks, particularly at higher doses.

The latest and most comprehensive analysis by the Cholesterol Treatment Trialists’ (CTT) Collaboration marked a significant advancement by using individual patient data rather than aggregated data[8]. This meta-analysis aimed to fill gaps in understanding about the size, timing, and risk factors for NOD with statin therapy.

The CTT meta-analysis included data from 19 trials comparing statin versus placebo and four trials comparing more versus less intensive statin therapy. This analysis involved over 123,000 participants with a median follow-up of approximately 4.5 years. The meta-analysis found that the proportional increase in risk was 10% for low- or moderate-intensity statin therapy (1.3% per year for statin vs. 1.2% per year for placebo) and 36% for high-intensity statin therapy (4.8% per year for statin vs. 3.5% per year for placebo). Among participants without diabetes at baseline, mean glucose and HbA1c levels increased slightly with statin therapy. Most new diabetes cases occurred in participants already at high risk, with baseline glycemic markers close to the diagnostic threshold for diabetes. This indicates that small increases in glycemia from statins are enough to push some high-risk individuals over the diagnostic threshold. For participants with diabetes at baseline, statin therapy also increased the risk of worsening glycemia. The relative risks were 1.10 for low- or moderate-intensity statin therapy and 1.24 for high-intensity treatment compared to placebo.

A significant bias identified was that participants in the statin treatment arms were likely monitored more closely for glycemic changes than those in the placebo arms. Similarly, monitoring was higher in high-intensity trials. This closer monitoring could lead to higher detection rates of NOD simply because of increased surveillance. The ‘surveillance effect’ implies that the more frequently you test, the more likely you are to detect abnormalities. In trials where HbA1c measurements were more frequent, the incidence of diabetes was higher, suggesting that part of the increased risk might be due to more rigorous monitoring rather than a direct effect of the statins. The extent of HbA1c measurement was a critical determinant of the observed absolute risk. The higher rates of new-onset diabetes in high-intensity trials could be attributed more to the frequency of HbA1c testing than to the inherent risk posed by the statin dose.

The study’s findings suggest that while statins modestly increase the risk of NOD, the absolute increase is small and predominantly affects individuals already at high risk. Therefore, NOD appears to be the result of a biochemical shift over the threshold classified for diabetes in large studies rather than an observation derived from clinical practice. Accordingly, the absolute changes in plasma glucose and HbA1c levels in patients without diabetes in the recent CTT were an increase in mean glucose by 0·04 mmol/L and in mean HbA1c by 0.06% with low-intensity or moderate-intensity statins. The majority of NOD cases were among participants who were already in the top quarter of the baseline distribution.

Despite these biases, the relative effects of statin therapy on NOD were consistent across different participant types and over time, supporting a genuine pharmacological effect rather than solely a monitoring artifact.

## NOD Risk with Other Agents

The IMPROVE-IT trial evaluated the risk of NOD with ezetimibe added to simvastatin versus placebo added to simvastatin in patients without diabetes at baseline[9]. This study involved 9500 patients, and NOD was defined based on initiation of antihyperglycemic medication or consecutive high blood glucose measurements. The trial found no significant difference in the incidence of NOD between the simvastatin/ezetimibe group and the simvastatin/placebo group[10]. The hazard ratio for NOD was 1.03 (95% CI, 0.93–1.15), indicating that the addition of ezetimibe to statin therapy did not increase the risk of developing diabetes.

The FOURIER trial, which studied the PCSK9 inhibitor evolocumab, assessed its efficacy and safety, including the impact on glycemia and the risk of developing diabetes[11, 12]. Evolocumab significantly reduced cardiovascular outcomes in patients with and without diabetes at baseline. Importantly, evolocumab did not increase the risk of new-onset diabetes in patients without diabetes at baseline, including those with prediabetes. The hazard ratio for new-onset diabetes was 1.05 (95% CI, 0.94–1.17). These findings suggest that evolocumab is safe in terms of diabetes risk while effectively lowering LDL cholesterol.

Inclisiran, an siRNA targeting PCSK9, was also evaluated for its effects on glycemia and diabetes risk. Phase 3 trials demonstrated that inclisiran provided substantial and sustained LDL cholesterol lowering across various glycemic and BMI strata, with no significant increase in the incidence of new-onset diabetes[13]. The incidence of NOD was comparable between the inclisiran and placebo groups, indicating a neutral effect on glycemia.

The CLEAR Outcomes trial investigated bempedoic acid, an ATP-citrate lyase inhibitor, in statin-intolerant patients[14]. This trial found that bempedoic acid significantly reduced the risk of major adverse cardiovascular events without increasing the risk of new-onset diabetes or worsening HbA1c levels[15]. The hazard ratio for NOD was 0.95 (95% CI, 0.83–1.09), demonstrating that bempedoic acid is a safe alternative for patients at risk of diabetes.

In consideration of this increasingly robust evidence from clinical trials and epidemiological studies that LDL-cholesterol lowering by statins can be associated in a dose-related manner with an increase in blood glucose levels, HbA1c, and risk for new-onset type 2 diabetes, a complex discussion has emerged about possible mechanisms. Therefore, we will address whether these observations are related to the direct mechanism of action of statins by looking at Mendelian randomisation analyses. Then we discuss potential mechanisms related to insulin resistance, being a key driver for the development of Metabolic Syndrome predisposing type 2 diabetes, and dysfunction in insulin secretion, playing a major role in an increase in glucose levels, and last but not least, manifestation of type 2 diabetes or NOD.

## Statins, Cholesterol Biosynthesis, and Diabetes: What Can We Learn From Genetics?

Table 1 summarizes clinical trials and genetic observations on the increase in glycemia between different LDL-cholesterol lowering agents and primary genetic alterations of respective drug targets. NOD appears to be somehow related to the activity of the LDL receptor-mediated uptake of LDL cholesterol into cells because patients with reduced LDL receptor activity seem to have a reduced diabetes risk, as discussed for patients with familial hypercholesterolemia [16]. The transport of statins into the liver is an unlikely candidate for diabetes risk because SLCO1B1 codes for a liver-specific organic anion transporter, and a rs4363657 variant is significantly associated with a higher risk for statin-induced myopathy. However, association of the C allele of SLCO1B1 rs4149056 was not associated with risk for type 2 diabetes, higher glucose concentrations, insulin resistance, or insulin secretion.

**Table 1:** Metabolic Effects of LDL-Lowering Agents and Genetic Targets

	Enhancing LDL Receptor Activity									
Pathway	Cholesterol synthesis pathway				Cholesterol absorption		LDL receptor degradation			
Target	ACLY		HMGCoA		NPC1L-1		PCSK9			
Mechanism of lowering	Genetically lower	BA	Genetically lower	Statins	Genetically lower	EZE	Genetically lower			PCSK9i MAbs
Efficacy										
LDL-C	Lower	Lower	Lower	Lower	Lower	Lower		Lower	Lower	
CVD	Lower	Lower	Lower	Lower	Lower	Lower		Lower	Lower	
Safety										
Weight or BMI	Lower	Lower	Higher	Higher	Unknown	Unknown		Unknown	Unknown	
HbA1c/glucose	Neutral	Neutral	Higher	Higher	Higher	Neutral		Higher	Neutral	
New-onset diabetes	Neutral	Neutral	Higher	Higher	Higher	Neutral		Higher	Neutral	

*ACLY* ATP-citrate lyase, *BA* bempedoic acid, *BMI* body mass index, *CVD* cardiovascular disease, *HMGCoA* 3-hydroxy-3-methylglutaryl coenzyme-A, *LDL* low-density lipoprotein, *LDL-C* low-density lipoprotein cholesterol, *MAb* monoclonal antibody, *NPC1L-1* Niemann–Pick C1-like 1, *PCSK9* proprotein convertase subtilisin/kexin type 9, *PCSK9i* proprotein convertase subtilisin/kexin type 9 inhibitor, *RCT* randomised controlled trial

Ray KK, et al. Lancet Diabetes Endocrinol (Suppl). 2024;12:19–28

As observed clinically with statin use, genetic data indicate that individuals with variations in the HMG-CoA reductase gene, which are associated with reduced enzyme activity, have an increased risk for type 2 diabetes [17]. In a recent large mendelian randomisation analysis with 654,783 participants, including 105,429 participants with major cardiovascular events, the ACLY and HMGCR scores chosen were associated with similar patterns of changes in plasma lipid and lipoprotein levels and accordingly had similar effects on the risk of cardiovascular events per decrease of 10 mg per decilitre in LDL cholesterol[18]. However, despite comparable LDL cholesterol levels, the observed risk for diabetes was neutral 0.97 (0.93–1.00) in the ATP citrate lyase group but increased 1.08 (1.05–1.12) in the HMG-CoA reductase group. This indicates that cellular metabolic pathways related to increased statin risk might not be primarily related to statin action in the liver. In accordance with that, clinical treatment with PCSK-9 antibodies or ezetimibe reducing LDL-cholesterol extracellularly or by liver selective action, like bempedoic acid or inclisiran, were neutral regarding diabetes risk [12, 13]. In addition, genetic variants affecting PCSK9 or the respective drug target of ezetimibe, which is the cholesterol transport protein Nieman Pick C1-like 1 protein (NPC1L1P), in all cells indicated increased diabetes risk, but not in respective clinical trials, where respective drugs target PCSK9 extracellularly or indirectly cholesterol metabolism of the liver. These discrepancies between clinical and genetic observations (table 1) and the fact that only statins are associated with increased diabetes risk in both clinical trials and genetic analyses indicate that NOD under lipid-lowering therapy with statins might be due to direct effects of statins in non-hepatic targets, like insulin-secreting beta cells. Statins are the only lipid-lowering molecules that can affect beta cells directly. Lipid-lowering treatment versus genetic target predisposition indicates that the statin-use-related diabetogenic mechanism appears to be intracellular.

Therefore, we will focus on statins, insulin resistance, and cellular cholesterol metabolism in the liver, followed by a discussion about the role of cellular cholesterol homeostasis and pancreatic insulin-secreting beta cells. Accordingly, there is accumulating evidence from clinical studies investigating the effects of statins on insulin resistance and/or insulin secretion that statins can affect both [16]. Recently, an open-label clinical trial of atorvastatin 40 mg daily in 71 adults without known atherosclerotic cardiovascular disease or type 2 diabetes at baseline investigated the effects on insulin resistance as assessed by steady-state plasma glucose during the insulin suppression test during the graded-glucose infusion test after 10 weeks[19]. Atorvastatin reduced LDL-cholesterol by 53% but did not change body weight. Compared with baseline, atorvastatin significantly increased insulin resistance (steady-state plasma glucose) by a median of 8%.

## Intracellular Cholesterol Synthesis in Liver and Risk for Diabetes: is It All?

Inhibition of cholesterol synthesis can lead to activation of cholesterol-dependent gene regulation (Table 2). The key transcription factors of lipid metabolism governing DNL are the sterol regulatory element-binding proteins (SREBPs), which were initially identified as cholesterol sensor for LDL receptor gene expression[20]. Two different genes are coding for SREBP-1 and SREBP-2 isoforms, whereas SREBF-1 is further transcribed into two splice variants, SREBP-1a and SREBP-1c [21]. While SREBP-2 mainly regulates cholesterol synthesis, the isoform SREBP-1c controls the synthesis of fatty acids. In contrast, the isoform SREBP-1a is involved in both pathways. Transcriptional inactive SREBP precursors are embedded in the endoplasmatic reticulum (ER) membrane as part of the intracellular endomembrane system. A complex proteolytic machinery releases the mature N-terminal transcriptional active SREBP domains, which translocate into the nucleus[22]. Since statins inhibit dose-dependent HMG-CoA reductase, they can activate SREBPs. Therefore, one question is whether activating SREBPs in the liver could drive diabetes manifestation. Transgenic overexpression of the constitutively active trans-domain of SREBPs in the liver of mice induces fatty liver disease. It has been shown that selective overexpression of SREBP-1 in the liver is associated with the induction of fatty liver disease and the development of severe obesity, ectopic lipid accumulation in skeletal muscle, and insulin resistance [23]. This development of clinical features in these transgenic mice corresponding to the metabolic syndrome was not related to increased food consumption, which was comparable between transgenic and control mice. Therefore, it appears that activation of SREBPs might increase gene regulatory susceptibility for the development of the metabolic syndrome and predisposition for type 2 diabetes. However, although liver metabolism and insulin resistance are risk factors for the development of type 2 diabetes, the clinical manifestation of NOD depends mainly on the degree of beta cell failure.

**Table 2:** Impact of Statins on Liver and Beta-Cell Function

Mechanism	Description
Liver	
Activation of SREBPs	Statins inhibit HMG-CoA reductase, leading to the activation of sterol regulatory element-binding proteins (SREBPs). Activated SREBPs can induce fatty liver, obesity, ectopic lipid accumulation, and insulin resistance.
Decreased Coenzyme Q10, Heme, and Isoprenoids	Statins reduce the production of coenzyme Q10, heme, and isoprenoid intermediates (FPP, GGPP), affecting small G protein function involved in cellular processes like cytoskeletal remodeling and secretory granule transport in beta cells.
Acetyl-CoA and FFA Receptors	Increased intracellular acetyl-CoA levels from HMGCR inhibition can produce acetate, activating FFA2 and FFA3 receptors on beta cells, reducing cAMP and insulin secretion.
ER Stress and Calcium Homeostasis	Cholesterol accumulation in the endoplasmic reticulum (ER) depletes calcium stores necessary for insulin release, induces ER stress, and activates the PERK-eIF2α pathway, leading to beta-cell injury and reduced insulin production.
Beta Cells	
LDL Uptake	LDLR-mediated LDL uptake into beta cells interferes with glucose-stimulated insulin secretion, potentially inhibiting insulin secretion through various pathways such as small G proteins, Ca2+ signaling, cell viability, and SNARE proteins.
Beta-Cell Dysfunction	Beta-cell-specific ablation of GGPP synthase and deletion of HMGCR lead to reduced beta-cell mass, insulin secretion, and glucose intolerance. Statins also inhibit mTOR signaling and small G protein genes, impairing beta-cell function.
Cholesterol Accumulation in Beta Cells	Overexpression of SREBP-2 in beta cells causes cholesterol accumulation, impairing insulin secretion. Excess cholesterol in beta cells disrupts granule trafficking, membrane protein distribution, and insulin granule exocytosis.
Trans-Golgi Network and Granule Trafficking	Cholesterol plays a key role in the trans-Golgi network for granule biogenesis and trafficking. Excess cholesterol disrupts granule formation and trafficking, impairing insulin release.
Lipid Rafts and SNARE Proteins	Cholesterol depletion in lipid rafts affects the spatial organization of SNARE proteins and channel proteins, impairing insulin granule exocytosis. Elevated cholesterol also reduces the density of voltage-gated Ca2+ channels.
Beta-Cell Apoptosis	Cholesterol accumulation in the plasma membrane induces beta-cell apoptosis, further impairing insulin secretion and contributing to diabetes development

## Beta Cells and Cholesterol Homeostasis: a Clue for Diabetes Risk?

Experiments in islets of wild-type and LDLR-knock-out mice, as well as comparative studies of hyperlipidemic mice with either LDLR knock-out or apoE knock-out, revealed that LDL receptor-mediated LDL uptake into beta cells interferes with glucose-stimulated insulin secretion[24]. Statins may reduce insulin secretion through various mechanisms, including disrupting the membrane association of small GTP-binding proteins, inhibiting glucose-induced cytosolic calcium signaling, compromising cell viability, and reducing the levels of SNARE (soluble N-ethylmaleimide-sensitive factor attachment protein receptor) proteins [25–28]. HMG-CoA reductase (HMGCR) controls not only de novo cholesterol synthesis but also the production of related intermediates, including farnesyl pyrophosphate (FPP) and geranylgeranyl pyrophosphate (GGPP)[29]. FPP and GGPP mainly regulate small G protein (sGP) farnesylation and geranylgeranylation, respectively; thus, FPP and GGPP modulate sGP function. The sGP family is involved in a wide range of cellular processes, such as cytoskeletal remodeling, vesicle fusion, and secretory granule transportation, which also occur in pancreatic islets. Beta-cell specific ablation of GGPP synthase results in b-cell dysfunction mediated by the deactivation of Rab27a, a Rab protein family member and sGP subtype. Accordingly, a recent study showed reduced pancreatic size and mass of beta-cells, fewer mature insulin granules, and reduced insulin secretion and glucose intolerance in mice treated with atorvastatin[29]. Further molecular analytics revealed that statin inhibited the expression of the pancreatic transcription factor, mechanistic target of rapamycin (mTOR) signaling, and sGP genes. Supplementation of the mevalonate pathway intermediate GGPP, produced by HMGCR, significantly restored the attenuated mTOR activity and beta-cell function after statin treatment.

To investigate directly the role of HMGCR in the development of beta-cells and glucose homeostasis, HMGRC was deleted in a beta-cell–specific manner by using the Cre-loxP technique[30]. Mice lacking HMGCR in beta-cells (b-KO) exhibited hyperglycemia and low insulin levels due to decreases in both beta-cell mass and insulin secretion. Histological analyses revealed dysmorphic islets with markedly reduced numbers of b-cells, some of which were also positive for glucagon. In conclusion, HMGCR plays a critical role in insulin secretion and beta-cell development in mice. According to the genetic studies and diabetes risk described above in Table 1, PCSK9-null male mice over 4 months of age showed less insulin in their pancreas[31]. These mice were hypoinsulinemic, hyperglycemic, and glucose-intolerant.

An additional effect of statins in beta cells might be that intracellular levels of acetyl-CoA might increase due to HMGCR inhibition. Acetyl-CoA can be a source for acetate production, an endogenous agonist of the G-protein-coupled receptors for short-chain fatty acids, FFA2 and FFA3, present on pancreatic beta cells. Both FFA receptors are coupled to Gαi, inhibiting adenylyl cyclase and thus reducing cAMP and, thereby, insulin secretion.

As discussed above, activation of SREBPs in beta cells by statins could also play a relevant role in altering insulin secretion and has been thoroughly reviewed previously [32]. Increased expression of SREBP-2 within beta cells leads to the accumulation of cholesterol, interfering with cellular function[33]. Under normal circumstances, inhibiting cholesterol biosynthesis activates SREBP2, boosts LDL receptor expression, increases cholesterol uptake, and reduces insulin secretion [34]. Similarly, disruptions in membrane transporters, such as ABCA1, responsible for clearing excess cholesterol, contribute to beta-cell dysfunction and a subsequent decline in insulin release [35].

Moreover, cholesterol accumulation in the ER can lead to calcium depletion, which is critical for insulin release, and trigger ER stress by activating the PERK-eIF2α pathway[36, 37]. Over time, this stress can result in beta-cell damage and reduced insulin production[38]. Additionally, cholesterol plays a key role in the trans-Golgi network, where it supports the formation and trafficking of secretory granules, essential for insulin storage, processing, and secretion in pancreatic beta cells[39]. Imbalances in cholesterol—either too much or too little—can disrupt insulin release by interfering with granule trafficking. Cholesterol depletion impairs granule formation, while excess cholesterol causes granule enlargement, distorts membrane protein distribution, and hinders the docking and fusion of granules with the plasma membrane.

Furthermore, cholesterol’s impact extends to beta-cell lipid rafts, which are vital for organizing SNARE proteins like syntaxin and SNAP25, as well as K+ ATP and voltage-gated Ca2+ channels[40]. Proper insulin secretion depends on the precise coordination between calcium influx and the exocytotic machinery. However, cholesterol depletion displaces SNARE and channel proteins from lipid rafts, disrupting insulin secretion. High cholesterol levels also reduce the density of voltage-gated Ca2+ channels, diminishing intracellular calcium flux and impairing the exocytosis of insulin granules. Excessive cholesterol in the plasma membrane is also linked to beta-cell apoptosis [36]. Collectively, these mechanisms underscore the importance of cholesterol metabolism in beta-cell function and suggest that its dysregulation could contribute to diabetes development.

## Conclusion and Perspective

**Fig. 1:**
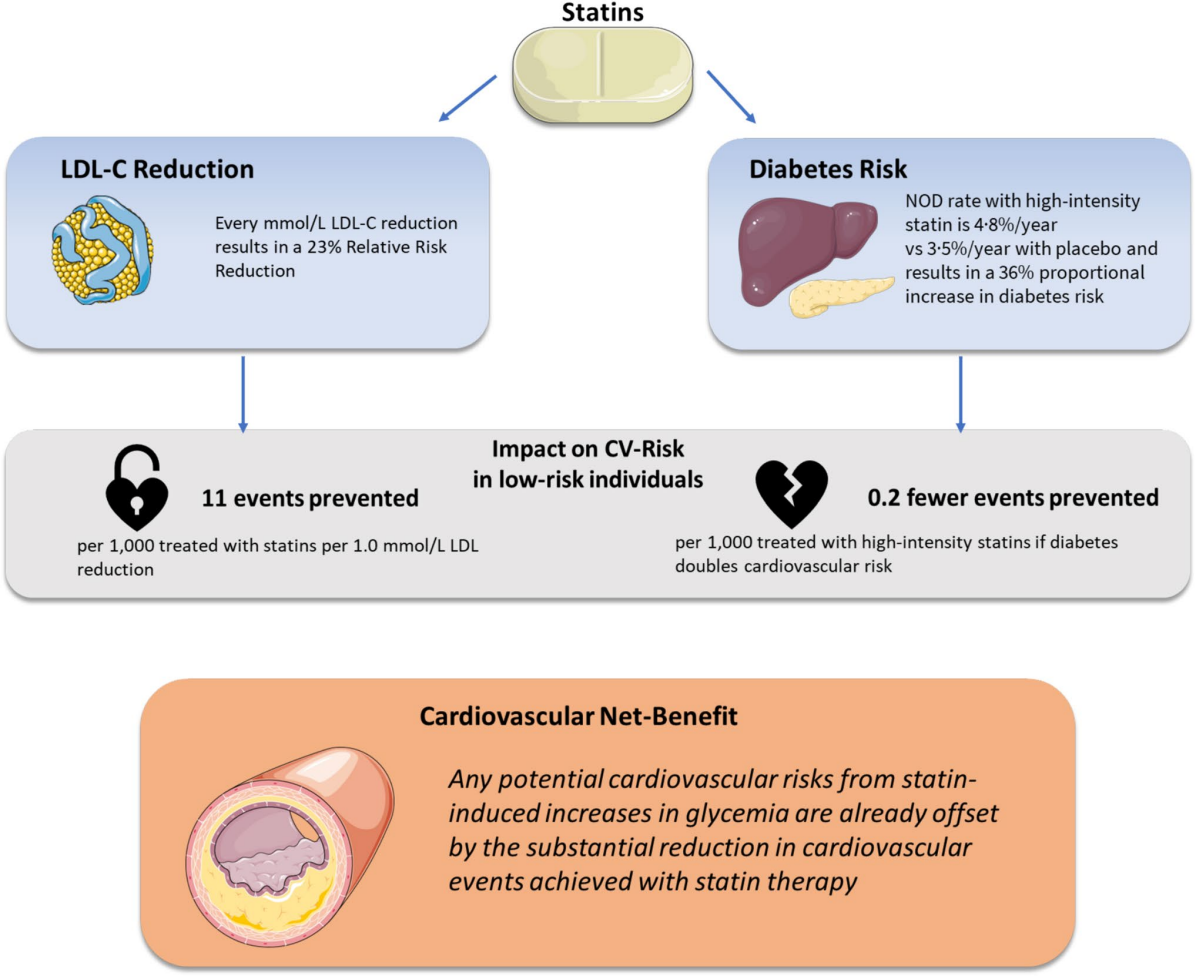
The net impact of statin therapy on cardiovascular risk versus the risk of new-onset diabetes (NOD)

From the clinical perspective, it is important to emphasize that the incidence of diabetes is defined by glycemia. Like other intermediate phenotypes, such as elevated blood pressure or lipid concentrations in plasma, diabetes becomes clinically relevant by its complications, i.e., CHD, nephropathy, retinopathy, and neuropathy. Statins also lower cardiovascular event rates in patients with manifest or latent diabetes, and post hoc analyses of trial data and registries indicate protective rather than harmful effects of statins on microvascular events. Overall, the risk/benefit ratio argues in favour of intensive LDL-C lowering despite the risk of increasing diabetes by shifting glucose levels above a biochemical threshold (Figure 1).

LDL receptor-related pathways modulated in the liver selectively by bempedoic acid, inclisiran, ezetimibe, and PCSk9 antibodies are clinically neutral in respect to NOD, which is in contrast to statin use. However, mendelian analyses indicate that not only HMGCR but also PCSK9 and NCP are potential candidates. Alterations in the candidates also modulate Beta-cell function, but only HMG-CoAR is affected by respective clinical treatment. This indicates that statin effects on beta cells might trigger the shift in glucose and HbA1c levels to the threshold defined by diabetes. This is in accordance with the clinical observation that statin-related NOD risk appears to be highest in individuals with prediabetes and relatively low insulin secretion.

Since prediabetes exhibits pathophysiological heterogeneity, one might identify specific subgroups with relevant diabetes risk modified by statin treatment in the future. Recently, a subgroup of individuals with prediabetes and the highest diabetes risk showed a cluster of obesity, insulin resistance, high levels of fatty liver, and low insulin secretion. The latter might predispose to statin-induced NOD due to the inherent dysfunction of beta cells and insulin secretion. In this case, statin treatment would correspond to a second-hit hypothesis to NOD.

## Key References

- Reith C, Preiss D, Blackwell L, Emberson J, Spata E, Davies K, et al. Effects of statin therapy on diagnoses of new-onset diabetes and worsening glycaemia in large-scale randomised blinded statin trials: an individual participant data meta-analysis. The Lancet Diabetes & Endocrinology. 2024;12(5):306-19. 10.1016/S2213-8587(24)-00040-8.
  - Recent meta-analysis using individual participant data, providing robust evidence on the relationship between statin therapy and new-onset diabetes, significantly advancing our understanding of the diabetogenic effects of statins.
- Shah NP, McGuire DK, Cannon CP, Giugliano RP, Lokhnygina Y, Page CB, et al. Impact of Ezetimibe on New‐Onset Diabetes: A Substudy of IMPROVE‐IT. Journal of the American Heart Association. 2023;12(13):e029593.
  - A study examining the impact of ezetimibe on new-onset diabetes, revealing no significant increase in risk when added to statin therapy.
- Leiter LA, Raal FJ, Schwartz GG, Koenig W, Ray KK, Landmesser U, et al. Inclisiran in individuals with diabetes or obesity: Post hoc pooled analyses of the ORION-9, ORION-10 and ORION-11 Phase 3 randomized trials. Diabetes, Obesity and Metabolism. 2024;26(8):3223-37. 10.1111/dom.15650.
  - Recent pooled analysis of Phase 3 trials on inclisiran, highlighting its efficacy and safety across glycaemic and BMI strata, showing no significant increase in new-onset diabetes risk.
- Ray KK, Nicholls SJ, Li N, Louie MJ, Brennan D, Lincoff AM, Nissen SE. Efficacy and safety of bempedoic acid among patients with and without diabetes: prespecified analysis of the CLEAR Outcomes randomised trial. The Lancet Diabetes & Endocrinology. 2024;12(1):19–28.
  - Subanalysis of the CVOT on bempedoic acid, demonstrating cardiovascular benefits without increasing new-onset diabetes risk, crucial for patients intolerant to statins.
- Ference BA, Ray KK, Catapano AL, Ference TB, Burgess S, Neff DR, et al. Mendelian Randomization Study of <i>ACLY</i> and Cardiovascular Disease. New England Journal of Medicine. 2019;380(11):1033-42. 10.1056/NEJMoa1806747.
  - Key Mendelian randomization study exploring the genetic impact of ACLY inhibition on cardiovascular disease and diabetes risk, showing a neutral diabetes risk profile compared to statins.
- Perego C, Da Dalt L, Pirillo A, Galli A, Catapano AL, Norata GD. Cholesterol metabolism, pancreatic β-cell function and diabetes. Biochimica et Biophysica Acta (BBA)-Molecular Basis of Disease. 2019;1865(9):2149-56.
  - A review highlighting the intricate connections between cholesterol metabolism and β-cell function, underlining the impact of cholesterol homeostasis on diabetes development.

## Data Availability

No datasets were generated or analysed during the current study.
